# Comprehensive Transcriptome Analyses of the Fructose-Fed Syrian Golden Hamster Liver Provides Novel Insights into Lipid Metabolism

**DOI:** 10.1371/journal.pone.0162402

**Published:** 2016-09-02

**Authors:** Ziyang Li, Chaoliang Xiong, Suo Mo, Haiying Tian, Mengqian Yu, Tingting Mao, Qian Chen, Haitao Luo, Quanzhen Li, Jianxin Lu, Yi Zhao, Wei Li

**Affiliations:** 1 Key Laboratory of Laboratory Medicine, Ministry of Education of China, School of Laboratory Medicine and Life Science, Wenzhou Medical University, Wenzhou, 325035, PR China; 2 Zhejiang Provincial Key Laboratory of Medical Genetics, Wenzhou Medical University, Wenzhou, 325035, PR China; 3 Key Laboratory of Intelligent Information Processing, Advanced Computer Research Center, State Key Laboratory of Computer Architecture, Institute of Computing Technology, Chinese Academy of Sciences, Beijing, 100190, PR China; 4 Department of Immunology & Microarray Core Facility, The University of Texas Southwestern Medical Center at Dallas, Dallas, TX, 75390, United States of America; Huazhong University of Science and Technology, CHINA

## Abstract

Dyslipidemia has been widely proven to contribute to cardiovascular diseases and other metabolic disorders, especially in insulin resistance and type 2 diabetes. The overproduction of VLDL is a significant characteristic of dyslipidemia, indicating the dysfunction of hepatic lipid metabolism, from triglyceride synthesis to transport. The fructose-fed Syrian golden hamster is an established animal model for the study of VLDL assembly with insulin resistance, however, it remains unknown how VLDL production is regulated at the transcriptional level due to the absence of a complete hamster genome. Here, we performed deep sequencing and constructed an mRNA-miRNA-lncRNA interaction network of Syrian golden hamster liver in order to reveal the global transcription profile and find potential RNA molecular regulation of VLDL production. We identified 4,450 novel multi-exon hamster lncRNAs and 755 miRNAs expressed in liver. Additionally, 146 differentially expressed coding genes, 27 differentially expressed lncRNA genes, as well as 16 differentially expressed miRNAs were identified. We then constructed an mRNA-miRNA-lncRNA interaction network that may potentially regulate VLDL production, and interestingly found several microRNA-centered regulatory networks. In order to verify our interpretation, miR-486 was selected for further experiments. Overexpression or down-regulation of miR-486 in fructose-fed hamsters resulted in altered hepatic expression of proteins involved in VLDL production, and in modulated levels of circulating VLDL. Our findings implicated that miR-486 is a potential regulator of circulating VLDL levels. These results provide new insights and a valuable resource for further study of the molecular mechanisms of VLDL secretion.

## Introduction

Cardiovascular disease (CVD) is a chronic disease which has become a serious threat to human health globally. Previous studies have indicated that patients suffering from CVD are often accompanied by hyperinsulinemia, which could increase the excessive secretion of VLDL, especially VLDL_1_[1]. Studies have verified that there are two main stages involved in the assembly of VLDL[2,3]: the first stage is lipid transfer to apoB with the regulation of the microsomal triglyceride transfer protein (MTTP) which forms the pre-VLDL particles that takes place at the endoplasmic reticulum (ER). The pre-VLDL particles then travel to the Golgi to undergo the second stage of VLDL assembly that requires a membrane transport step [4,5]. The second stage is the pre-VLDL maturation following the fusion of the pre-VLDL particles with triglyceride droplets which involves further lipidation and processing. The mature VLDL particles are then secreted. Molecular mechanisms mediating hepatic VLDL overproduction in the insulin resistance state have been partially verified, such as the overexpression of PTEN[6,7] and PTP-1B[8].The reduced insulin action increase FoxO1 activity, subsequentlyinduces MTTP protein facilitating VLDL assembly and induces apoCIII reducing peripheral triglyceride catabolism[9].

Hamster has been developed as a model used in the study of VLDL assembly, as similar with humans, only apoB-100 is synthesized in hamster livers, which is different from other rodents such as mouse and rat with both apoB-48 and apoB-100 produced in livers [10, 11]. It was found that the fructose-fed Syrian golden hamster exhibited a typical whole body insulin resistance with marked hepatic VLDL and TG overproduction, which was set up for an ideal model investigating VLDL assembly in insulin resistance[12].

Mechanisms mediating lipid metabolic regulation at transcriptional level have been attracting increasing attention [13]. Therefore, it has become necessary to further elucidate the transcriptional regulation mechanisms involved in VLDL synthesis. Recent studies have been revolutionarily changed due to the rapid development of next-generation sequencing (NGS) technologies, which provide new platforms for comprehensive transcriptional studies in animals, plants, and microbes[14]. In recent years, noncoding RNAs, such as microRNAs and long noncoding RNAs (lncRNAs) have increasingly emerged as important in mammalian transcriptomes [15–17]. LncRNAs, mRNAs and miRNAs can interact with each other in the regulatory networks of metabolism. Numerous studies have indicated that lncRNAs are an important component in fundamental biological processes such as reprogramming[18], X-chromosome inactivation[19,20], epigenetic regulation[21], and transcriptional regulation[22]. However, despite a handful of well-studied lncRNAs, such as Xist, HOTAIR, and H19 [23–25], the biological functions of the majority of lncRNAs remain unknown. It was assumed that the changes in miRNA and lncRNA expression in the fructose-fed Syrian golden hamster model could contribute to lipid metabolism variations. Thus, identifying lncRNAs and miRNAs and analyzing their changes in Syrian golden hamster liver is antecedent.

However, one stumbling block that remains is the unfinished status of the Syrian golden hamster genome sequencing, which is still underway at the Broad Institute (NCBI Bioproject 77669 and 210213). This introduces complications when utilizing this model to investigate VLDL molecular processes at the transcriptome level. More recently, Schmucki.*et al*[26] and Tchitchek.*et al*[27] performed Syrian golden hamster transcriptome analysis based on *de novo* transcriptome assembly. In this study, based in part on the transcriptome annotation of these two groups, we are intending to reveal the differentially expressed coding genes, lncRNAs, and miRNAs, which might be involved in the synthesis of VLDL. This work will provide novel insights into the molecular mechanisms of VLDL synthesis, and contribute to the prevention and treatment of lipid metabolic disorders. To the best of our knowledge, this is the first description of whole-transcriptome patterns in the liver of the fructose-fed Syrian golden hamster model.

## Results

### Transcriptome Reconstruction and Classification

**Fig 1:**
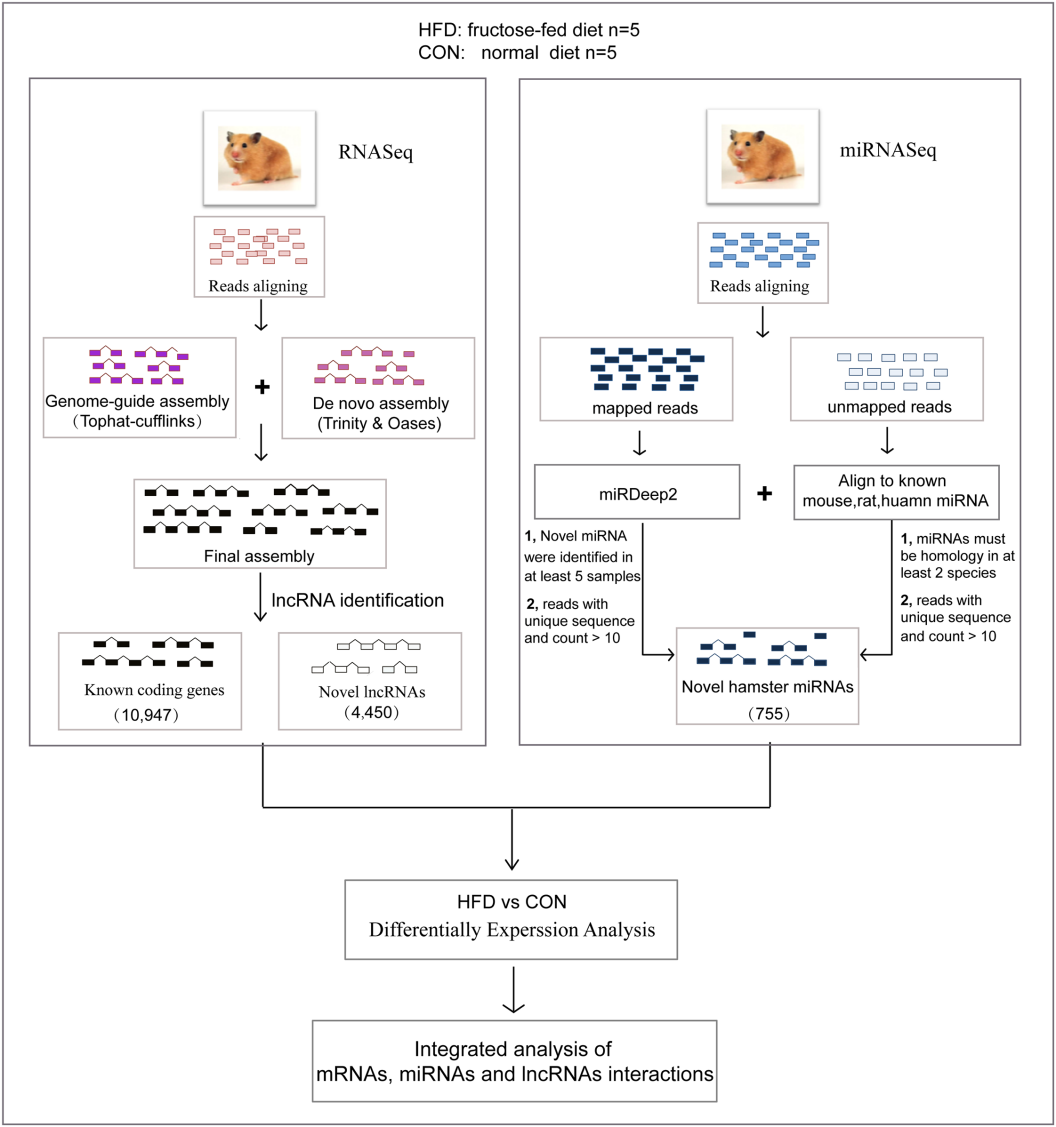
Overview of the data processing pipeline.

Following our data processing pipeline (Fig 1), a total of 257.02 million clean reads were mapped to the reference genome MesAur1.0 with short-read gapped aligner TopHat version 2.0.6[28], which recovered 203.8 million (79.29% average mapping rate) mapped reads S1 Table). Then using our transcriptome reconstruction strategy (S1 Fig, S2 Table), we obtained 103,473 transcripts at 65,240 gene loci which covered 10,947 known coding genes.

### Hamster lncRNA Identification

To date there have been no lncRNAs identified in Syrian golden hamster. However, as 117,405 lncRNAs were identified in the mouse genome according to the NONCODE database [29], there might be a similar number of lncRNAs in the hamster genome. Due to the fact that our sequencing data came from unstranded library protocols, we focused solely on intergenic and intronic lncRNAs. Based on the features of lncRNAs reported in vertebrates[30], the identification of lncRNAs here were performed following a strict work flow[31,32] (S2 Fig). We found a total of 4450 lncRNA transcripts from 3454 gene loci in the hamster genome (S3 Table).

**Fig 2:**
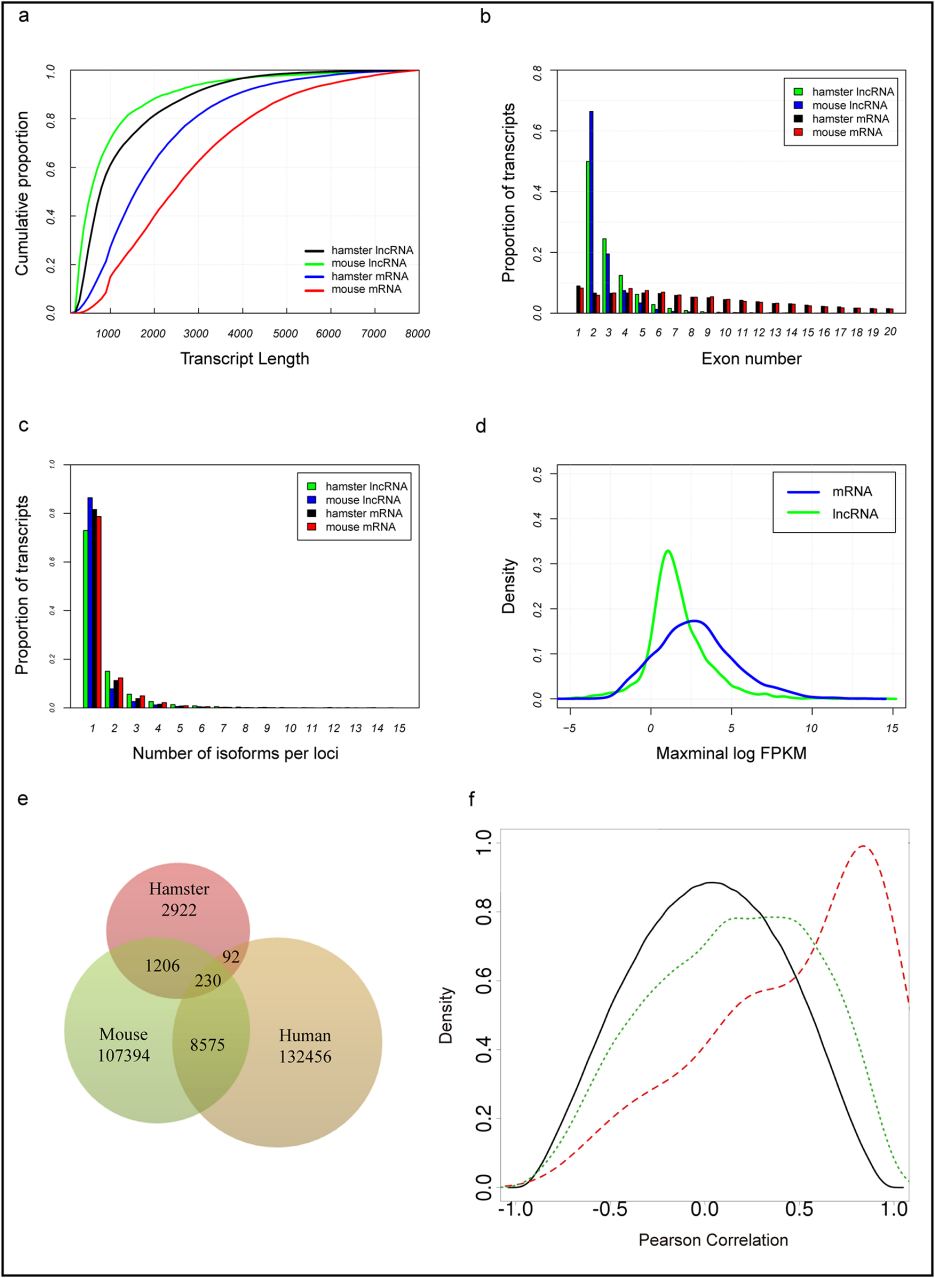
Basic features and conservation of hamster lncRNAs. (a) The cumulative distributions of transcript lengths for the hamster lncRNAs (green), hamster protein-coding genes (blue), mouse lncRNAs (black), and mouse protein-coding genes (red). (b) The distribution of exon numbers for hamster lncRNAs (green), hamster protein-coding genes (blue), mouse lncRNAs (black), and mouse protein-coding genes (red). (c) The distribution of the number of isoforms identified for hamster lncRNA genes (green), hamster protein-coding genes (blue), mouse lncRNA genes (black), and mouse protein-coding genes (red). (d) The expression levels of hamster lncRNAs were lower than hamster protein-coding genes. Maximal expression abundance (log2-normalized FPKM counts as estimated by Cufflinks) of each lncRNA (blue) and protein-coding gene (green). (e) Sequence conservation of lncRNAs between hamster and other mammals. (f) Correlation analysis of hamster lncRNA and coding gene pairs. Black: random lncRNA-coding gene pairs. Green: neighbouring lncRNA-coding gene pairs. Red: different expression neighbouring lncRNA-coding gene pairs.

To characterize the basic features and conservation of hamster lncRNAs, we first analyzed the genomic features of our identified lncRNAs, with comparison to hamster coding transcripts, and mouse lncRNAs and coding transcripts (Fig 2). The overall lengths of the identified novel lncRNA transcripts (1117 nt, on average) was shorter than hamster protein-coding transcripts (2064 nt, on average) (Fig 2a), and the exon number of the novel lncRNAs (2.7 exons, on average) tended to be lower than hamster coding transcripts (10.3 exons, on average) (Fig 2b). Our identified hamster lncRNAs were also alternatively spliced (Fig 2c). Additionally, the average expression levels of novel hamster lncRNAs were lower compared with protein coding genes, while the lncRNAs also had a wider range of abundance (Fig 2d), which was consistent with the basic features of lncRNAs reported in mouse.

Then, we aligned the identified hamster lncRNA transcripts with those of human and mouse lncRNAs using BLASTN version 2.2.29+ with an E-value ≤ 10^−5^ to identify sequence conservation of lncRNAs between hamster and other mammals. The NONCODE database contains 141,353 human lncRNAs and 117,405 mouse lncRNAs. In total, 328 (7.37%) and 1436 (32.27%) of the hamster lncRNAs had detectable homology with human and mouse lncRNAs, respectively, while 230 (5.17%) shared conservation between human and mouse. In comparison, 8805 (7.50%) mouse lncRNAs had a similar detectable homology with human lncRNAs following our criteria (Fig 2e). The features of our identified hamster lncRNAs shared similar genomic features and homology features with human and mouse lncRNAs, indicating that they are *bona fide* hamster lncRNAs.

Several studies have indicated that some lncRNAs may act in *cis* to regulate the gene expression of their chromosomal neighborhood [33]. To explore whether hamster lncRNAs were transcribed coordinately with their neighbouring coding genes, and examine the regulatory roles of the hamster lncRNAs, we analyzed hamster lncRNAs that were located within 20 kb of protein-coding genes. In total, 2102 hamster lncRNA genes were located within 20 kb from at least one protein-coding gene, and 3201 lncRNA:coding gene pairs were found. We then analyzed the correlation expression patterns between neighbouring lncRNA:coding gene pairs, differently expressed neighbouring lncRNA:coding gene pairs, and random lncRNA:coding gene pairs with Pearson correlation analysis (Fig 2f). We observed that the correlated expression patterns of differently expressed neighbouring lncRNA:coding gene pairs was higher than all neighbouring lncRNA:coding gene pairs. Moreover, neighbouring lncRNA:coding gene pairs exhibited a more correlated expression pattern compared to random lncRNA:coding gene pairs. This observation suggested that hamster lncRNAs tended to be transcribed coordinately with their neighbouring coding genes, especially among the differentially expressed neighbouring lncRNA:coding gene pairs.

### Hamster miRNA Identification and Annotation

After filtering out low-quality reads and removing the adaptor sequences, a total of 70.96 million clean reads were obtained. 52.91 million (74.57%) clean reads were mapped to the reference genome. In total, 359 novel hamster miRNA precursors were predicted by miRDeep2 (Fig 1; S3 Fig). Then we manually checked the conservation of these novel miRNA with other species (mouse and human). 220 novel pre-miRNAs homologous to known miRNAs in mouse and human were detected and named as “mau-mir-xxx” (“mau” is short for Mesocricetus auratus). The remaining 139 novel pre-miRNAs may be hamster-specific novel miRNAs. Due to the incompleteness of the hamster genome, the unmapped reads matched 37 homologous mature miRNAs in other species with reads count no less than 10. Altogether, we identified 755 mature miRNA sequences (S4 Fig; S4 Table).

### High Fructose-Fed Alter Intrahepatic Gene Expression

**Fig 3:**
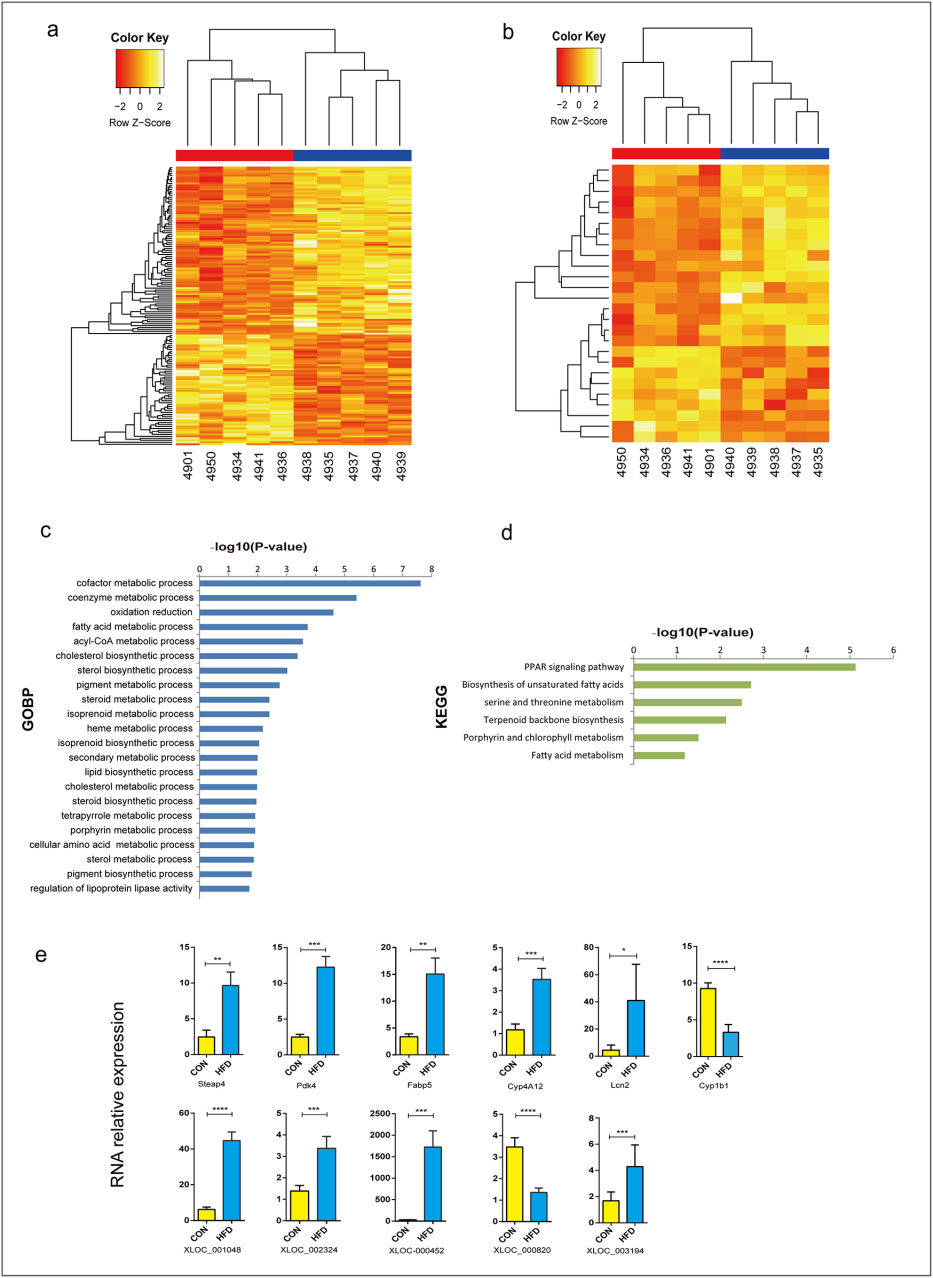
Differentially expressed liver protein-coding genes and lncRNAs. (a) Differentially expressed liver protein-coding genes. (b) Differentially expressed liver lncRNA genes. (c) GO terms (biological processes) of the identified differentially expressed coding genes. (d) KEGG pathway analysis of the identified differentially expressed coding genes. (e) qPCR results for coding genes and lncRNAs. *(Data were analyzed by the Δ Δ Ct method*, *the values represent the means with SD*, *n = 6)*.

In our RNASeq data from high fructose vs. normal diet, a total of 146 significant differentially expressed coding genes and 27 lncRNA genes were identified, with a criteria of at least a 2 fold difference and a p-value less than 0.05 (p-value ≤ 0.05 and |log2FC| ≥ 1) (Table 1; S5 Table). Hierarchical clustering analysis was used to depict the significant (p-value ≤ 0.05) differentially expressed coding genes and lncRNAs between the two groups (Fig 3a and 3b). Among the 146 differentially expressed coding genes, 86 were up-regulated and 60 down-regulated. Gene Ontology and pathway analysis showed that some of these differentially expressed coding genes were involved in lipid metabolic processes, glucose metabolic processes, hexose metabolic processes, monosaccharide metabolic processes, oxidation reduction, oxidoreductase activity, the PPAR signaling pathway, fatty acid metabolism, and biosynthesis of unsaturated fatty acids (Fig 3c and 3d; S6 Table). Most genes related to fatty acid metabolic processes (such as Cyp4a10, Hacl1, Ehhadh, Abhd5, Acot11, Fads2, Acot5, and Acot4), and glucose metabolic processes (such as Gck, Pdk4, and Fabp5) were up regulated in the fructose-fed hamsters. These results indicated that high fructose feeding may increase the VLDL secretion through lipid metabolic processes and glucose metabolic processes.

**Table 1:** Number of differentially expressed mRNAs, miRNAs and lncRNAs.

Comparison	Differentially expressed genes (P<0.05)		
	Total	Up	Down
**Coding genes**	**146**	**86**	**60**
**lncRNAs**	**29**	**17**	**12**
**miRNAs**	**16**	**10**	**6**

To further validate these differentially expressed genes, real-time RT-PCR analysis was performed randomly on 8 mRNAs and 6 lncRNAs. The results revealed that most of the selected genes (6 mRNAs and 5 lncRNAs), were successfully validated (Fig 3e). The high confidence of the validation indicated that most differentially expressed mRNAs and lncRNAs in our study were truly expressed in the fructose-fed Syrian golden hamster.

### A High Fructose Diet Altered Intrahepatic miRNA Expression

**Fig 4:**
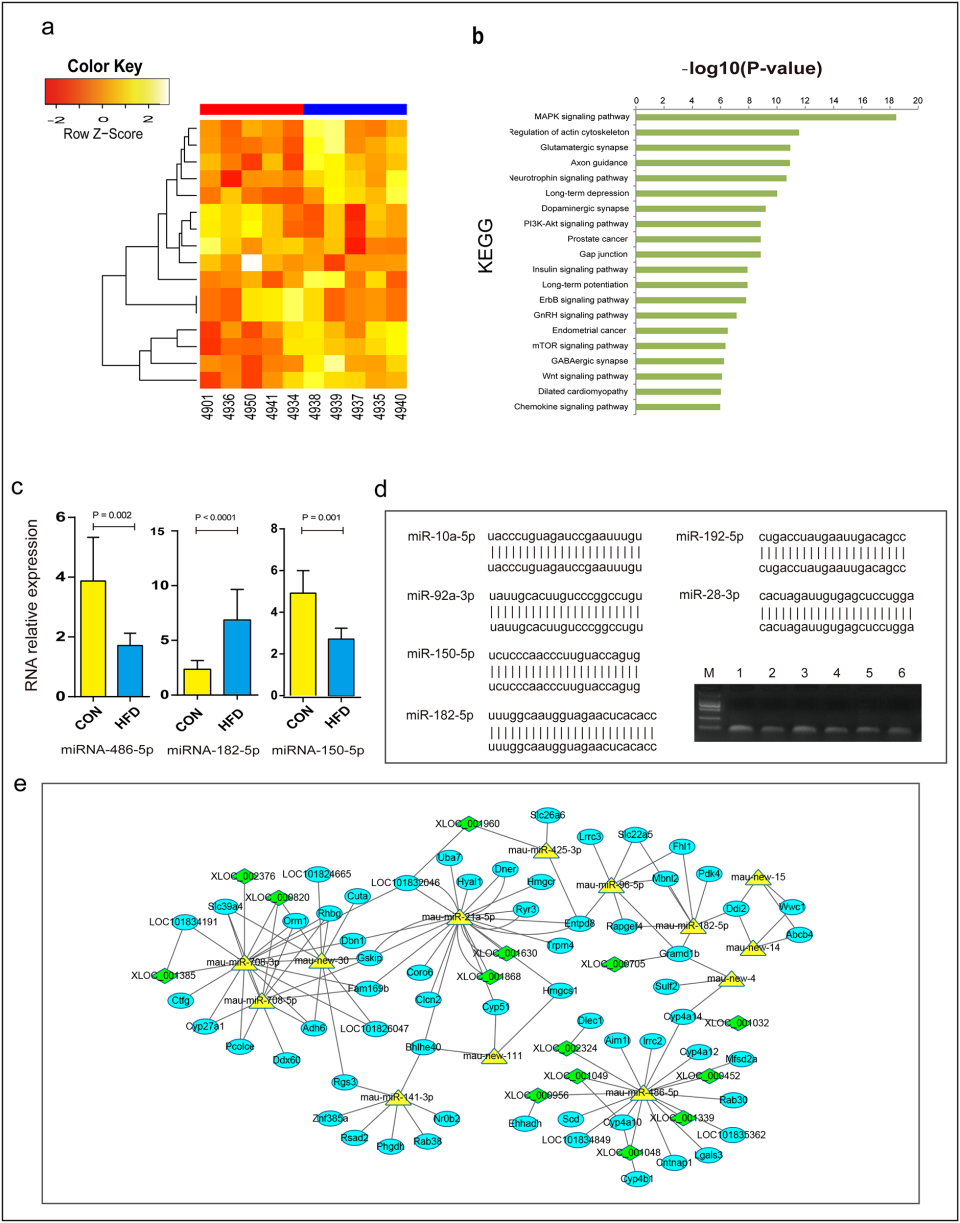
Differentially expressed liver miRNAs and the interaction network. (a) Differentially expressed liver miRNAs. (b) KEGG pathway analysis of the identified differentially expressed miRNAs. (c) qPCR results for miRNAs. *(Data were analyzed by the Δ Δ Ct method*, *the values represent the means with SD*, *n = 10)*. (d) Validation of selected miRNAs. miRNAs from M to 6: marker (DL 2000), miR-10a-5p, miR-28-3p, miR-92-3p, miR-150-5p, miR-182-5p, miR-192-5p. (e) Differentially expressed mRNAs, miRNAs and lncRNAs interactions on VLDL secretion. This interaction network diagram was made by using Cytoscape.

In our miRNA-sequencing data, a total of 16 miRNAs were differentially expressed with a threshold of at least a 1.5 fold difference and a p-value less than 0.05 (p-value ≤ 0.05 and foldchange ≥ 1.5) (Fig 4a; Table 1; S7 Table). Among these 16 miRNAs, 10 were up-regulated and 6 down-regulated. Five of these differentially expressed miRNAs were hamster-specific novel miRNAs.

To explore the biological functions of these differentially expressed miRNAs, we predicted the potential target genes of these miRNAs. Altogether, 2293 unique genes (S8 Table) were found. Through miRNA pathway analysis of these miRNAs, 50 KEGG pathways were found (p < 0.05) (Fig 4b; S9 Table). Among the 50 KEGG pathways, some were involved in metabolic processes, such as the MAPK signaling pathway, the PI3K-Akt signaling pathway, the Insulin signaling pathway, and the mTOR signaling pathway.

To validate the differentially expressed miRNAs identified by sequencing, miR-486, miR-182, miR-183 and miR-150 were selected for qRT-PCR analysis. Three of the selected miRNAs showed significant differences between the two groups, confirming the results of miRNA-seq. To further verify the predicted novel miRNAs, we randomly selected 6 miRNAs for PCR amplification, cloning, and sequencing. The results showed that all of them fully matched with the predicted genes (Fig 4c and 4d).

### Integrated Analysis of mRNA, miRNA and lncRNA Interactions on Lipid Metabolism

To further illustrate the differentially expressed mRNAs, miRNAs and lncRNAs interactions on VLDL assembly and secretion, we first predicted the target genes of the miRNAs and the lncRNAs target miRNAs. We then analyzed the correlated expression patterns between differentially expressed mRNAs, miRNAs and lncRNAs. Finally, we integrated the mRNA-miRNA, mRNA-lncRNA, miRNA-lncRNA, and mRNA-mRNA interactions by using Cytoscape (Fig 4e). The interactions with negative correlations for miRNA-mRNA and miRNA-lncRNA were selected. In these miRNA-mRNA-lncRNA interactions, several miRNAs highly interacted with multiple mRNAs and lncRNAs, such as miR-486, miR-182, miR-141, miR-21, and miR-708. Studies have indicated that miR-21[34], miR-182[35], and miR-141[36] are related to lipid metabolism and cardiovascular disease. Expression levels of miR-486 (log2cpm = 13.7 across all samples) and miR-182 (log2cpm = 10.1 across all samples) were relatively high.

### MiR-486 Regulates the VLDL Level in Hamster Serum

**Fig 5:**
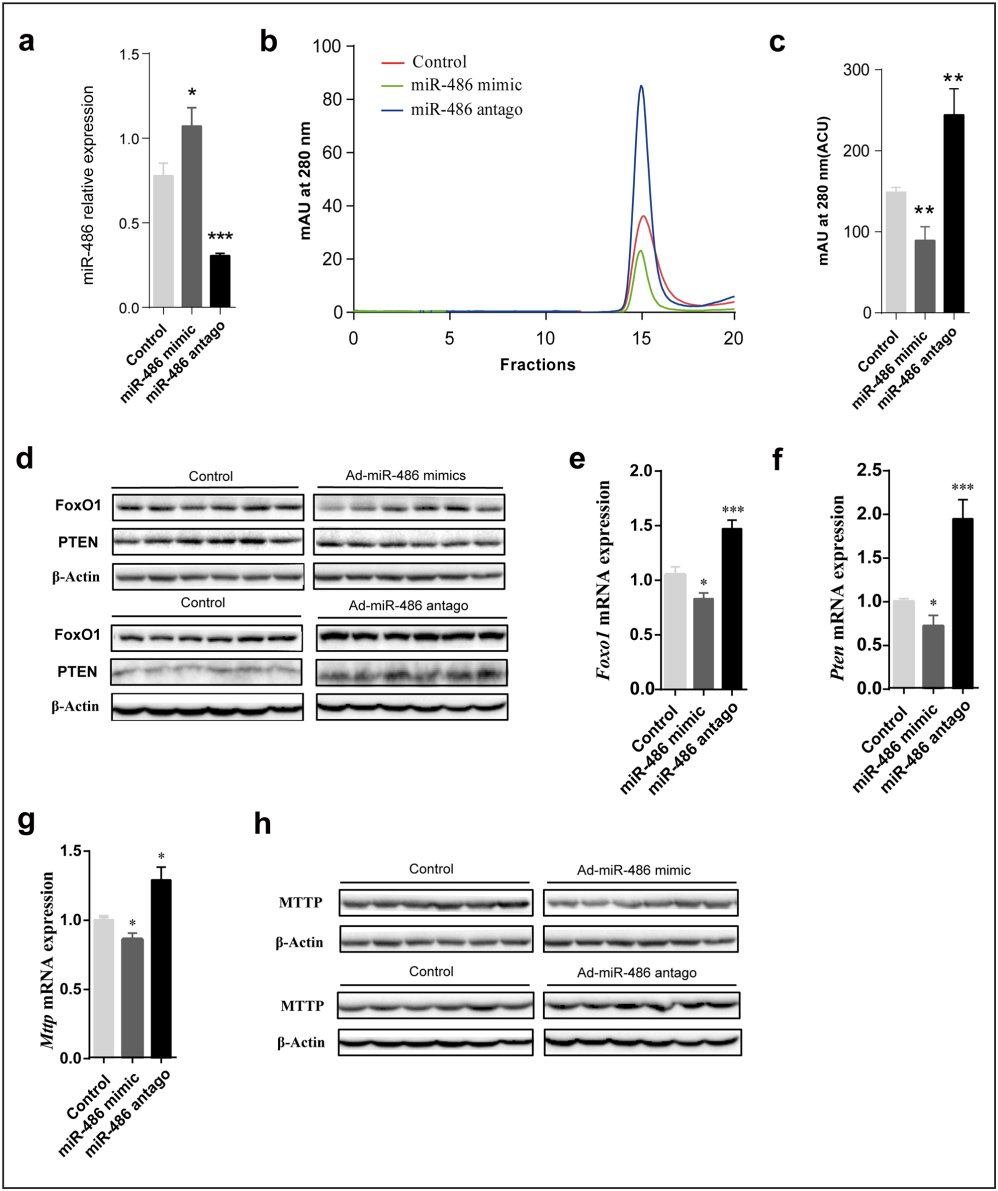
miR-486 regulates the production of VLDL through the PI3K-Akt-Signaling Pathway by targeting PTEN and Foxo1a. (a) Hepatic miR-486 levels determined by quantitative RT-PCR in hamsters injected with Adenovirus harboring miR-486 mimics and miR-486 antago, as well as a scrambled control. n = 6 per group. (b) Fast protein liquid chromatography (FPLC) analysis of sera from fructose-fed Syrian golden hamsters (n = 6) injected with lentivirus expressing miR-486. (c) Mean area under the curve (AUC) values calculated for VLDL fractions 14–16 isolated by FPLC from b. (d, h) Immunoblot analysis of hepatic Foxo1, Pten (d), and Mttp (h) in liver tissue from fructose-fed hamsters treated with miR-486 mimics and miR-486 antagonist showing expression levels of Foxo1. β-actin was used as a loading control. (e-g) Hepatic expression of Foxo1 (e), Pten (f), and Mttp (g) in hamsters injected with Adenovirus harboring miR-486 mimics and miR-486 antagonist, as well as a scrambled control. n = 6 per group.

Interestingly, a recent study[37] indicated that phosphatase and tensin homolog (PTEN) and Foxo1a, which negatively affects phosphoinositide-3-kinase (PI3K)/Akt signaling, are the targets of miR-486. Overexpression of miR-486 will reduce PTEN and Foxo1a protein levels, which, in turn, enhances PI3K-Akt signaling. Based on these findings, we hypothesized that miR-486 may regulate the production of VLDL through the PI3K-Akt-signaling pathway by targeting PTEN and Foxo1a. We transfected fructose-fed Syrian golden hamsters with miR-486 mimics and miR-486 antagonist to explore whether miR-486 had an effect on circulating blood VLDL *in vivo*. Hamsters were injected in the orbital vein with adenovirus expressing miR-486 mimics or miR-486 antagonist, or were injected with a control virus, and then serum and livers were harvested after two weeks. As shown in Fig 5a, 5b and 5c, compared with controls, the VLDL levels in hamster serum was decreased by transfection with miR-486 mimic, while it was increased by treatment with miR-486 antagonist. Further, we evaluated the mRNA and protein levels of Foxo1a, PTEN and MTTP in hamster livers transfected with miR-486 mimic and antagonist. Our results showed that miR-486 overexpression reduced hepatic levels of Foxo1a, PTEN, and MTTP compared with controls. On the other hand, inhibition of miR-486 increased hepatic levels of Foxo1a, PTEN, and MTTP compared with controls. Together, our findings suggested that miR-486 regulated circulating VLDL levels in the fructose-fed Syrian golden hamster model, probably through the PI3K/Akt-signaling pathway by targeting PTEN and Foxo1a.

## Discussion

Previous studies have shown that various mechanisms are responsible for VLDL production and secretion under insulin resistance [38]. Howerver, these mechanisms are mainly at the translational and post-translational levels mediated by the insulin signaling pathway, and little is known regarding the molecular mechanisms of the transcriptional regulation of VLDL production.

In our study, lipid droplet-associated protein genes, such as *Cidec*, *Plin1*, *and Plin2*, were up-regulated in fructose-fed hamsters. *Cidec* (cell death-inducing DFF45-like effector C), which belongs to the cide family that includes *Cidea*, *Cideb*, and *Cidec*, was significantly highly expressed (with a fold change of 2.5) in the fructose-fed hamster liver. Cideb, an ER- and Lipid Droplet-Associated Protein, is reported to be localized to the surface of lipid droplets and the ER, and also interacts with apoB-100/-48 and increases VLDL lipidation[39,40]. Studies have indicated that the expression of Cidec will potentially promote lipid droplet formation, TG-accumulation, and impair insulin sensitivity in hepatocytes[41]. Cidec is also localized to the surface of lipid droplets [42]. In addition, Cidec has been identified as a PPARγ target and is a direct regulator of PPARγ-dependent hepatic steatosis[43]. Plin1 (also called Perilipin1), a member of the PAT family, was highly expressed in the fructose-fed hamster liver, and is also reported to interact with Cidec to promote lipid droplet size in human adipocytes [44]. Studies have also shown that up-regulated expression of Plin2 promotes lipid accumulation and lipid droplet formation in various cells [44–46]. Thus, these lipid droplet-associated protein genes may play a critical role in VLDL lipidation and maturation. Taken together, our results provide strong evidence that the overproduction of VLDL in Syrian golden hamsters may be partly due to *de novo* lipogenesis (DNL) and cholesterol synthesis.

As lncRNAs are known to interact with chromatin proteins to positively and negatively regulate expression of their neighboring genes [47], lncRNAs that were located within 20 kb of a protein-coding gene were selected for analysis. We found a lncRNA gene, XLOC_001048, located in the intron of LOC101844652 (Cyp4a10) (S5 Fig) where the correlation coefficient of XLOC_001048 and Cyp4a10 was 0.998. This lncRNA gene exhibited a 4.23 fold change in the fructose-fed hamster, while Cyp4a10 was also up-regulated with a 3.26 fold change. In the present study, fatty acid oxidation was regulated by Cyps (*Cyp 2E1*, *4A10* and *4A14*) [48]. This observation implies that XLOC_001048 may be a regulatory element for Cyp4a10, and that XLOC_001048 may, together with Cyp4a10, contribute to the overproduction of VLDL in fructose-fed hamsters. The biological functions of the overwhelming majority of lncRNAs remain unknown, therefore, further experimental studies are needed to illuminate the functions of lncRNA genes and to clarify the mechanisms of the production and secretion of VLDL in the fructose-enriched diet hamster.

It was also interestingly found that multiple miRNA moleculars could potentially be the modulators through the interaction network analysis. In this study, miR-486, which has been proved to target PTEN and Foxo1a was selected for the further verification.miR-486 mimic and antagonist were used to investigate the functions of miR-486 on VLDL. Enhancement of miR-486 function decreased VLDL secretion, whereas reducing miR-486 increased VLDL secretion. These findings strongly implicated that miR-486 may regulate the production of VLDL by controlling the expression of the PTEN, FoxO1a, and MTTP proteins, and suggested a potential theraputic strategy for dyslipidemia.

## Conclusions

RNASeq analysis of fructose-fed Syrian golden hamster provided us with some interesting novel insights into lipid metabolism. From the transcriptome analysis, we found 146 significant differentially expressed coding genes, 26 differentially expressed lncRNA genes, and 16 differentially expressed miRNAs. GO and pathway analysis revealed that the overproduction of VLDL in fructose-fed Syrian golden hamsters was regulated by genes involved in multiple metabolic processes. Our experimental studies have shown that miR-486 may modulate the production of VLDL by regulating the expression of the PTEN, FoxO1a, and MTTP proteins. This was the first study examining transcriptome data and differential expression in the fructose-fed hamster, and provided essential information for further studies investigating novel molecular mechanisms of VLDL synthesis. In addition, the newly identified 4450 lncRNAs and 755 miRNAs will also facilitate the functional study of lncRNAs and miRNAs in this species.

## Materials and Methods

### Animal Model Building

All experimental and surgical procedures involving Syrian golden hamsters (*Mesocricetus auratus*) were approved by the Institutional Animal Care and Use Committee of the Wenzhou medical University (Wenzhou, China) via the accession number wydw2014-0128. The fructose-fed Syrian golden hamster model building protocol was utilized according to the procedures previously described [12]. All animals were housed in pairs and given free access to food and water. After blood collection, animals were given either the control diet (normal chow) or the fructose-enriched diet (hamster diet with 60% fructose). The diet was continued for 2 weeks and hamster weight was monitored every 2 days. According to Guidelines for Endpoints in Animal Study Proposals, all Syrian golden hamsters were euthanized (exsanguinated while under sedation) and samples were harvested from each hamster. After complete general anesthesia was achieved, the livers were perfused as described [49]. Liver tissues were then excised and rinsed with 1×PBS, subdivided in freezing tubes, and preserved in liquid nitrogen at -196°C. All operations were performed on ice.

### cDNA Library Preparation and Sequencing

Liver tissues were homogenized into a fine powder before RNA extraction. Total RNA was extracted using the RNeasy Mini Kit (Qiagen) according to the manufacturer’s instructions, including treatment with DNase. RNA quality was evaluated with an Agilent 2100 Bioanalyzer (Agilent RNA 6000 pico & DNA 1000 Kit). All RNA samples had an RNA integrity number (RIN) no less than 7.5. Sequencing libraries were prepared using the Illumina TruSeq RNA-Seq library protocol coupled with a Poly-A protocol according to the manufacturer’s instructions. For miRNA sequencing, the Illumina TruSeq Small RNA-Seq library protocol was used. After quality control, all the libraries were sequenced on a high-throughput sequencing platform-Illumina HiSeq^™^ 2000. The raw reads have been uploaded on the NCBI-SRA database and are available via the BioProject PRJNA320146.

### Reads Processing, Transcriptome Assembly and Classification

For the comprehensive analysis of the whole transcriptome of fructose-fed Syrian golden hamster livers, we applied a pipeline for the data processing: 1) Transcriptome reconstruction: a genome-guide transcriptome assembly was combined with a *de novo* transcriptome assembly strategy. 2) Comparison of the reconstructed transcriptome with the reference gene annotations. 3) LncRNA identification using our pipeline and stringent criteria. 4) Downstream analysis: differential expression analysis and functional analysis. Details are described in S1 Supporting Methods

### LncRNA Identification Pipeline

To identify novel reliable hamster lncRNAs, the following pipeline and criteria were used (S1 Supporting Methods; S3 Fig). To derive a more comprehensive hamster liver transcriptome, the identified hamster lncRNAs and known RefSeq genes expressed in the hamster liver were integrated by Cuffcompare. This uniform hamster liver transcriptome was used to calculate transcript expression levels. Details are described in S1 Supporting Methods.

### Hamster miRNA Identification Pipeline

The low quality short reads and 3' adapter from the deep-sequencing reads were filtered out generating a non-redundant Fasta format via a Perl script. Only sequences with a length longer than 18 nt were retained for further analysis S3a and S3b Fig). The reads were aligned to the reference hamster genome using bowtie with default parameters. The remaining reads that did not map to the draft genome were then aligned to all known miRNAs in the miRbase to detect homologous miRNAs in other species. The genome-mapped reads were then used for prediction of new hairpin precursors and mature miRNAs expressed in hamster liver tissue using miRDeep2. Authentic novel miRNAs should be detected in multiple samples (at least 5 out of the 10 samples) with reads >10, the miRDeep2 score cutoff was set to 1 and both had the mature and star sequences. We then aligned the predicted hairpin precursors and mature miRNAs to all known mouse and human miRNAs to determine whether the novel miRNAs have homologous genes in other species.

### Potential Function Prediction of Differentially Expressed miRNAs

DIANA miRPath (v.2.0) was used to predict potential target genes and pathways of the significant differentially expressed miRNAs between the two groups. Because hamster genes were not contained in the current version of DIANA miRPath, mouse miRNAs were used for function prediction. The P-value threshold was 0.05 and the MicroT threshold was 0.8.

### Gene Expression Analysis

Cuffdiff v2.0.2[28], a tool provided by Cufflinks, was used to detect the differentially expressed genes (both coding and lncRNAs) between the two groups with default settings for all parameters. For miRNA differential expression, edgeR was applied. The identified miRNAs read counts were normalized using a rescaling factor computed as the proportion of the total amount of reads mapped to these novel miRNAs. Comparisons were accepted to be significant at a p-value of 0.05.

### Functional Analysis

Gene Ontology and pathway functional enrichment analysis was carried out based on the differentially expressed genes using the DAVID bioinformatics database, which was used to perform gene function enrichment analysis via Gene Ontology (GO) and the Kyoto Encyclopedia of Genes and Genomes (KEGG), a Benjamini correction of p-value ≤ 0.05 was used as the criterion for enrichment[50]. Enrichment analysis based on Hypergeometric distribution was used to identify the significantly enriched functional classification or metabolic pathways in differentially expressed genes. Cytoscape was used to identify the potential networks of differentially expressed coding genes, miRNAs, and lncRNAs[51].

### Quantitative RT-PCR

To validate the sequencing results, quantitative real-time PCR was conducted using an Applied Biosystems 7500 Real-Time PCR System (Applied Biosystems, Foster City, CA, USA). After extraction, total RNA and miRNA were reverse transcribed using the High Capacity cDNA Reverse Transcription Kit. 10 differentially expressed genes were randomly selected to confirm using the SYBR PremixExTaq^™^ protocol (TaKaRa). Primers used in the quantitative real-time PCR were designed using Premier 5.0 (Table 2). Each sample was performed in triplicate under the following amplification conditions: 95°C for 10min initially, and then 40 cycles of 95°C for 15 s and 60°C for 1min. The efficiency of quantitative real-time PCR was defined using the standard curve method. Quantification of mRNA was performed using the average cycle thresholds (Ct). 18S rRNA was used as an endogenous control gene to evaluate the gene expression levels.

**Table 2:** Primer pairs selected for validation by qRT-PCR.

Genes	Primer sequence	
	Forward (5' -3')	Reverse (5'-3')
18S rRNA	**TAAGTCCCTGCCCTTTGTACACA**	**GATCCGAGGGCCTCACTAAAC**
Lcn2	**ATGGAGGTGACATTGTAGTTGG**	**CAGTTTCAGGGGAGGTGGTA**
Steap4	**TGATGTATGGCGGCAAGCG**	**GCACGGCATTATGAGGATGAGC**
Fabp5	**GCTCTTCGGAAAATGGGTGCC**	**AAACTGCGTCGTCTTCAAAGTGC**
Pdk4	**GCCCCTTTGGCTGGTTT**	**AGATGATGGCGTCTGTCCC**
Cyp4a12	**CAAGTGCCTGCCCACATTG**	**TCTCCATTCAACAGAAGCAAACCA**
Foxo1	**CATGAACCGATTGACCCC**	**GATACCCATCCTACCATAGCC**
Pten	**CTGCACGAATAATAAGGCAT**	**AAATTGAAGCCCTAATCCC**
Mttp	**ATGCTGACCTTTGTGCGAGA**	**GTGGGCAACCATCTCCTTGA**
XLOC-001048	**AGTGCTGAACAAAATGTGACCG**	**TGTGGAGCTTCTACTTCTTTAGGG**
XLOC-000452	**CCACCTCAGGGAATGAACCA**	**CCCCACTGCAAGAGTAAACCTAG**
XLOC-000820	**TCACGGGTGGTACATTGTGG**	**AGGAGGACGAGGTGGAAAAGA**
XLOC_002324	**GCGGTTCAGCATTTCACGGC**	**AGGCAGGAGGCTAGTCAACACTT**

### MiR-486 Mimics and Inhibitor Transfection

Adenovirus harboring miR-486 mimics and miR-486 antagonist, as well as a scrambled control, were obtained from Hanbio, Inc. 6-week-old male Syrian golden hamsters were purchased from the Charles River Laboratory, Beijing, China. The fructose-fed Syrian golden hamster model was built as previously described. The fructose-fed hamsters were injected in the orbital vein with 6 × 10^10^ pfu/hamster diluted in 75 μl PBS on day 1, 1.5 × 10^10^ pfu/hamster diluted in 50 μl PBS on day 7, and serum and liver were harvested on day 14.

### VLDL Analysis by Fast Performance Liquid Chromatography (FPLC)

Equal volumes of serum samples were pooled from male Syrian golden hamsters from three groups in the fasting states at the end of 2 weeks of adenovirus injection. Lipoproteins were separated using fast protein liquid chromatography (FPLC) on a Superose 6 10/300 GL column (GE Healthcare Bio-Sciences AB, Uppasala, Sweden). Samples were chromatographed at a flow rate of 0.5 ml/min, and fractions of 500 μl each were collected. Mean area under the curve (AUC) values were calculated for VLDL fractions 14–16 isolated by FPLC.

### Western Immunoblot Analysis

Proteins from male Syrian golden hamster liver samples was extracted with RIPA buffer [50mmol/l Tris-HCl (pH 7.4), 1% NP-40, 0.5% sodium deoxycholate, 150 mmol/l NaCl, 0.1% SDS, EDTA] containing protease and phosphatase inhibitors. Protein concentrations were measured using a BCA-100 Protein Quantitative Analysis Kit. After denaturation, protein samples were subjected to SDS-PAGE and blotted onto polyvinylidene difluoride membranes (Millipore). Nonspecific binding sites were blocked with 5% skim milk in Tris-buffered saline containing 0.1% Tween 20 for 1h and then incubated with primary antibodies against MTTP (Affinity), PTEN, β-Actin (Beyotime Biotechnology), Akt, Akt-phospho-Ser473 (Cell Signaling Technologies), Foxo1a, or Apolipoprotein B (Abcam) overnight at 4°C. After three washes in Tris-buffered saline containing 0.1% Tween 20 the membranes were incubated with horseradish peroxidase conjugated secondary antibodies (anti-mouse or anti-rabbit IgG) for 1h and visualized by ECL detection (Bio-Rad). Quantitation was performed by Image J software.

## Supporting Information

S1 Fig Transcriptome reconstruction pipeline. (TIF)

S2 Fig LncRNA identification pipeline. (TIF)

S3 Fig Characteristics of miRNA sequencing reads. (a) Length distribution of one of the ten samples. (b) Classification of the sequenced reads (one of the ten samples).
(TIF)

S4 Fig Predicted secondary structures of several novel hamster miRNAs. (TIF)

S5 Fig Characteristics of XLOC_001048 lncRNA. The tracks depict transcripts assembled by Cufflinks and RefSeq gene annotations; Right-to-left arrows indicate transcripts on the minus strand.
(TIF)

S1 Supporting Methods Additional methods for transcriptome assembly and lncRNA identification. (DOCX)

S1 Table Sample information and mapping statistics. (XLSX)

S2 Table Transcriptome reconstruction statistics. 1, Genome-guide transcriptome assembly statistics. 2, De novo assembly statistics.
(XLSX)

S3 Table Identified hamster novel lncRNAs. (XLSX)

S4 Table Identified hamster novel miRNAs. 1,Conservative hamster novel miRNAs. 2, Hamster-specific novel miRNAs.
(XLSX)

S5 Table Differential expressed coding genes and lncRNAs. 1,Differential expressed coding genes. 2, Differential expressed lncRNAs.
(XLSX)

S6 Table Gene Ontology and pathway analysis of DE coding genes. (XLSX)

S7 Table Differential expressed miRNAs. (XLSX)

S8 Table Potential target genes of differentially expressed miRNAs. (XLSX)

S9 Table KEGG pathways of genes targeted by DE miRNAs. (XLSX)
