# Supplementary figures and images for: Comprehensive Transcriptome Analyses of the Fructose-Fed Syrian Golden Hamster Liver Provides Novel Insights into Lipid Metabolism
Source: PLoS One. 2016 Sep 2;11(9):e0162402. doi: 10.1371/journal.pone.0162402 (PMC5010245; doi:10.1371/journal.pone.0162402)

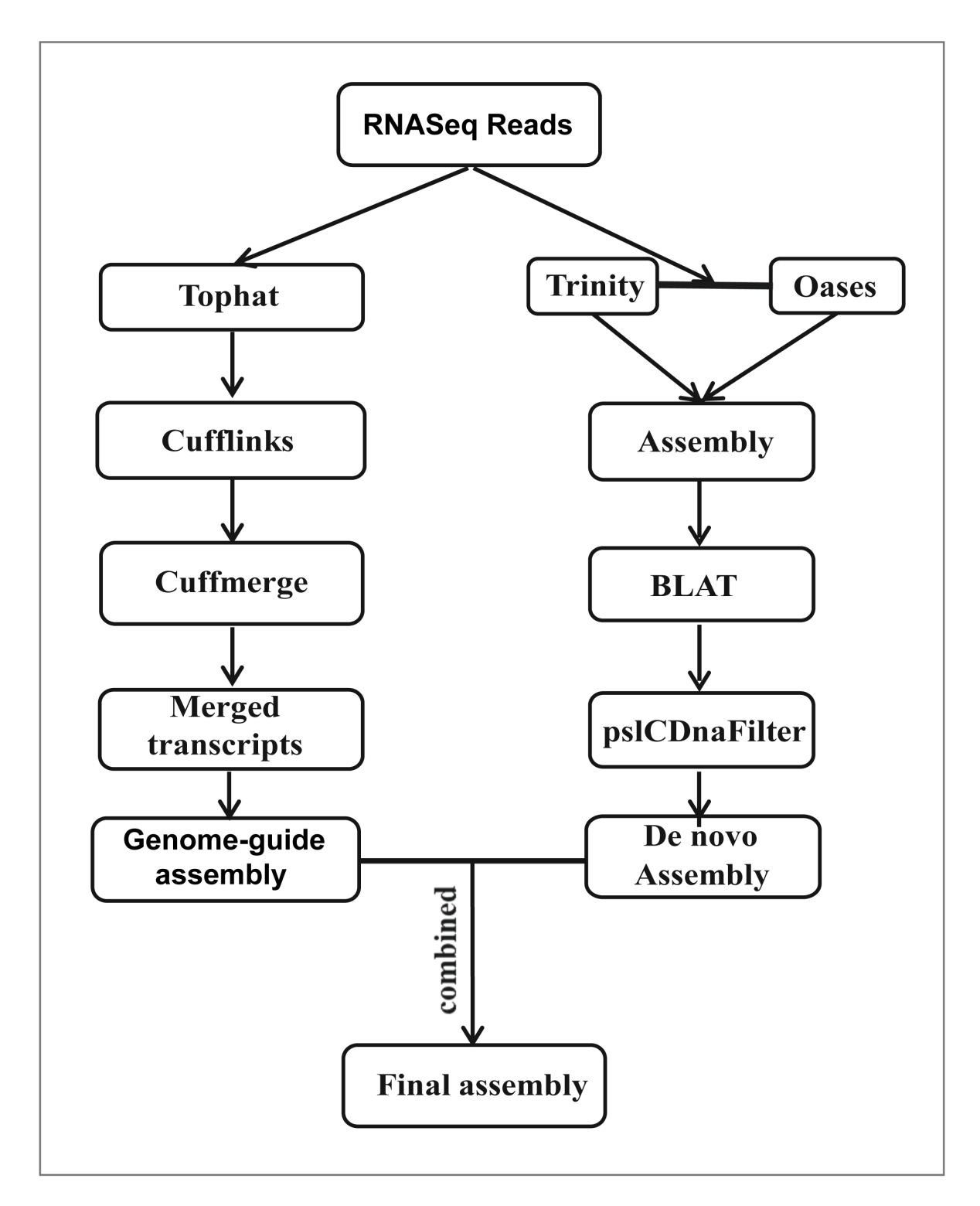

Supplement: S1 Fig — (TIF) [file pone.0162402.s001.tif]

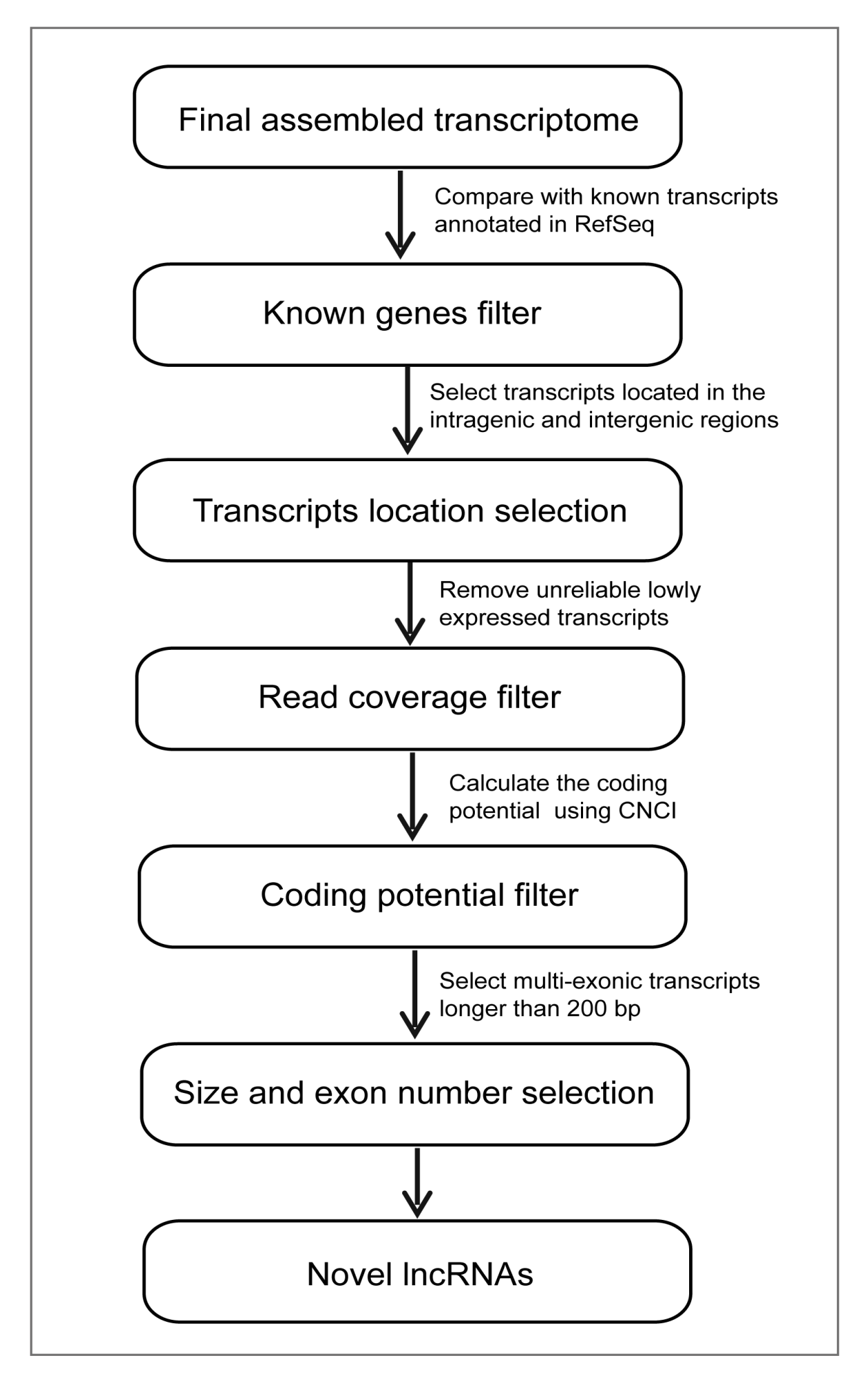

Supplement: S2 Fig — (TIF) [file pone.0162402.s002.tif]

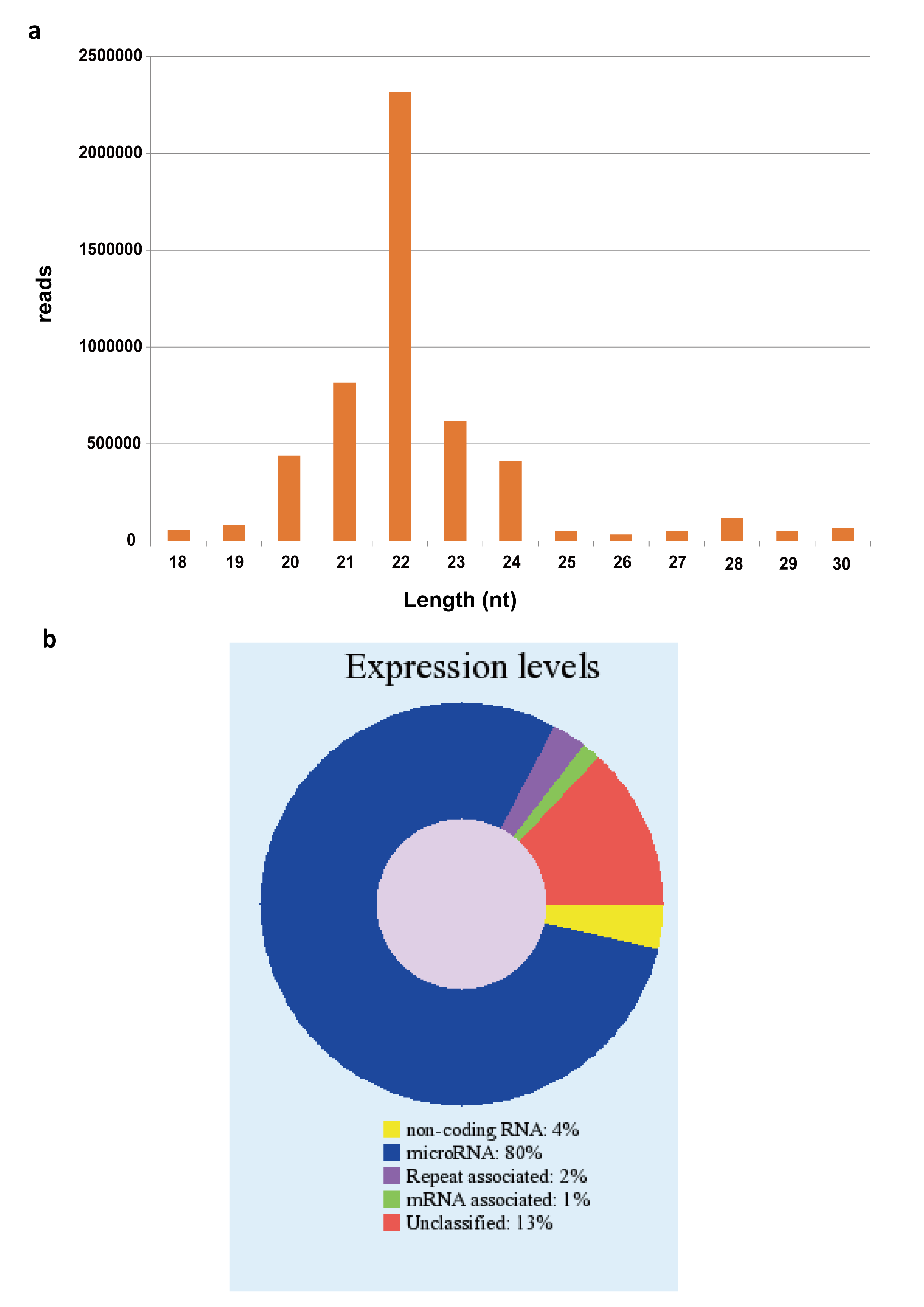

Supplement: S3 Fig — (a) Length distribution of one of the ten samples. (b) Classification of the sequenced reads (one of the ten samples). (TIF) [file pone.0162402.s003.tif]

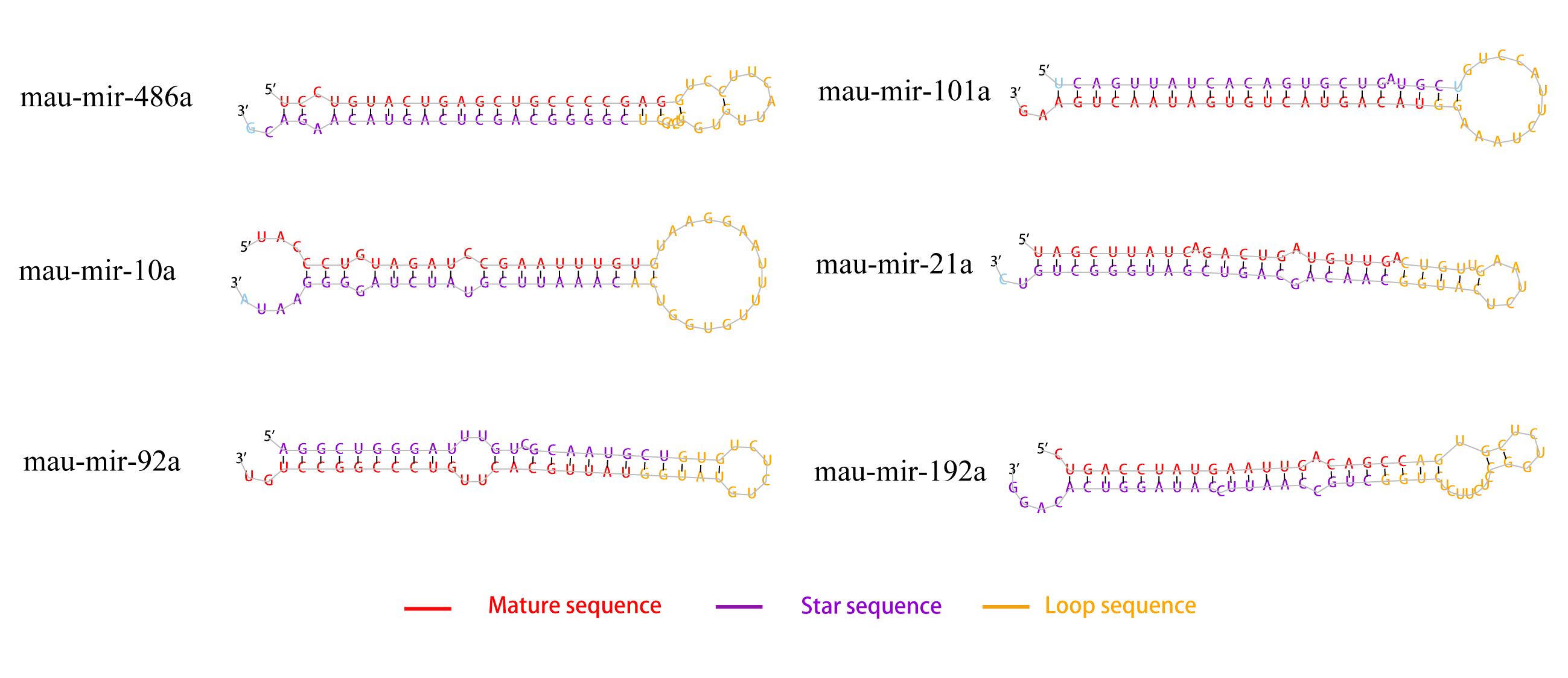

Supplement: S4 Fig — (TIF) [file pone.0162402.s004.tif]

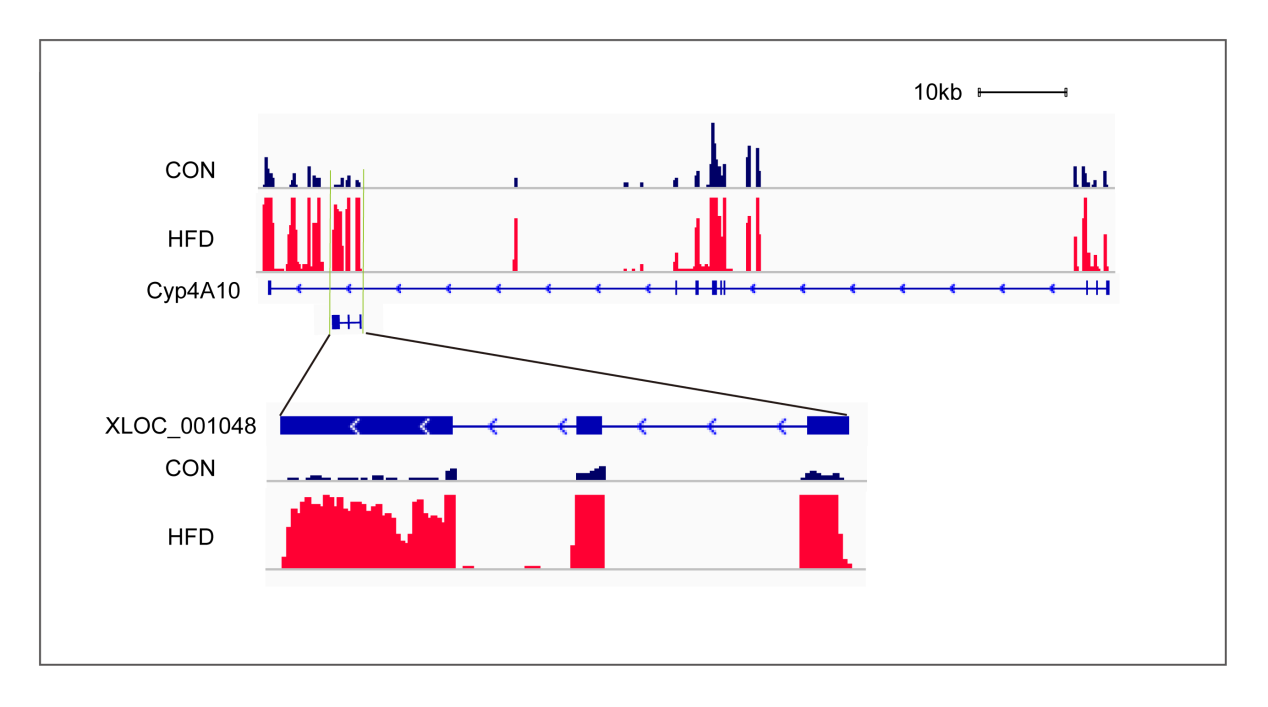

Supplement: S5 Fig — The tracks depict transcripts assembled by Cufflinks and RefSeq gene annotations; Right-to-left arrows indicate transcripts on the minus strand. (TIF) [file pone.0162402.s005.tif]
