# Supplementary material for: Comprehensive Transcriptome Analyses of the Fructose-Fed Syrian Golden Hamster Liver Provides Novel Insights into Lipid Metabolism
Source: PLoS One. 2016 Sep 2;11(9):e0162402. doi: 10.1371/journal.pone.0162402 (PMC5010245; doi:10.1371/journal.pone.0162402)
Supplement: S1 Supporting Methods — (DOCX) [file pone.0162402.s006.docx]

**Comprehensive transcriptome analyses of the fructose-fed Syrian golden hamster liver provides novel insights into lipid metabolism**

Ziyang Li^1^, Chaoliang Xiong^1^, Suo Mo^1^, Haiying Tian^1^, Mengqian Yu^1^, Tingting Mao^1^, Qian Chen^1^, Haitao Luo^3^, Quanzhen Li^4^, Jianxin Lu^1,2,*^, Yi Zhao^1,3,*^, Wei Li^1,2,*^

1 Key Laboratory of Laboratory Medicine, Ministry of Education of China, School of Laboratory Medicine and Life Science, Wenzhou Medical University, Wenzhou 325035, PR China

2 Zhejiang Provincial Key Laboratory of Medical Genetics, Wenzhou Medical University, Wenzhou 325035, PR China

3 Key Laboratory of Intelligent Information Processing, Advanced Computer Research Center, State Key Laboratory of Computer Architecture, Institute of Computing Technology, Chinese Academy of Sciences, Beijing 100190, PR China.

4 Department of Immunology & Microarray Core Facility, The University of Texas Southwestern Medical Center at Dallas, Dallas, TX 75390, USA

*Correspondence: [biozy@ict.ac.cn](mailto:biozy@ict.ac.cn;) (Yi Zhao); liweiwzmc@163.com (Wei Li); [jxlu313@163.com (Jianxin Lu)](mailto:jxlu313@163.com;)

**Supporting Methods**

**Transcriptome assembly**

Reads were aligned to the Syrian golden hamster draft genome MesAur1.0 using TopHat[[1](#_ENREF_1)], a program specifically designed to align RNA-seq reads and discover *de novo* splice junctions, with the default settings. For the transcriptome reconstruction, we applied a strategy that combined a genome-guide transcriptome assembly method and a *de novo* assembly method for the transcriptome assembly (Supplementary Fig S2). The genome-guide *ab initio* assembler was TopHat-Cufflinks[[2](#_ENREF_2)] (with the default settings and‘min-frags-per-transfrag = 0’), which robustly and reliably reconstructed both coding and noncoding transcripts based on the partial assembled genome. Our *de novo* strategy utilized two different assemblers: one was the single k-mer assembler Trinity, which is reported to do well in recovering full-length transcripts and spliced isoforms [[3](#_ENREF_3), [4](#_ENREF_4)]. This assembly was performed with default parameters. The second one was the multi-kmer assembler Velvet-Oases[[5](#_ENREF_5)], which explored a range of k-mer lengths to generate an overall assembly covering wide transcript expression levels. The k-mer length was set to values of 27, 29, 31, 33 and 35 for different sub-assemblies. Then these sub-assemblies were then merged into one. CD-HIT-EST v.4.6[[6](#_ENREF_6)] (with parameters ‘-c=0.9; -n=8’) and CAP3[[7](#_ENREF_7)] (with default parameters) were used to merge the assemblies generated from Trinity and Oases. Oases tends to be more sensitive, Trinity more accurate[[5](#_ENREF_5)]. This *de novo* strategy found novel transcripts that the genome-guide assembler could not find [[8](#_ENREF_8), [9](#_ENREF_9)]. After that, we aligned the merged *de novo* assembled transcriptome to the Syrian golden hamster genome sequence using blat[[10](#_ENREF_10)](with parameters ‘-q=rna, -minIdentity=90, -minMatch=2), and pslCDnaFilter (downloaded from UCSC, with the following parameters: minQSize=20; minNonRepSize=16; ignoreNs; bestOverlap minId=0.95; minCover=0.80 ) was used to filter the blat results. We then combined the two transcriptomes using the Cuffcompare module provided by Cufflinks. Finally, a more complete transcriptome was generated. This strategy considered the partial reference genome and allowed sequence reads to be mapped to chromosome regions or transcripts not yet annotated in the predicted reference annotation. In summary, the genome-guide and *de novo* strategies were combined to create a more comprehensive transcriptome, which took advantage of the high accuracy and sensitivity of the genome-guide assemblers, while leveraging the ability of the *de novo* assemblers to identify novel transcripts, combining the two complementary strategies[[8](#_ENREF_8), [11](#_ENREF_11)].

We then compared the final assembly with the reference coding gene annotations using Cuffcompare, allowing the assemblies to be classified into the following categories: (1) known coding genes (transcripts with class code “=jc” according to Cufflinks when compared with the coding genes); (2) known noncoding genes (e.g. pseudogenes, miscellaneous RNAs, tRNAs, or rRNAs) (transcripts with class code “=jc” according to Cufflinks when compared with the known noncoding genes); (3) potentially novel transcripts (transcripts with class code “iu” according to Cufflinks) and; (4) undefinable transcripts (transcripts with other class codes according to Cufflinks). The potentially novel transcripts might be novel coding or noncoding transcripts for downstream analysis.

**LncRNA** **identification pipeline**

(1) Compare the final assembled transcriptome with the annotated genes in RefSeq using the Cuffcompare module provided by Cufflinks; (2) Filter for known annotations: remove transcripts that overlapped with hamster protein-coding genes, pseudogenes, miscellaneous RNAs, tRNAs, or rRNAs obtained from NCBI RefSeq (NCBI Bioproject 210213) and keep the remaining transcripts that are located in the intragenic and intergenic regions; (3) Remove unreliable lowly expressed transcripts: We ran Cufflinks with its transcript abundance calculation mode to estimate the read coverage of each transcript across the 10 samples. At FPKM of about 0.3, RNA-seq reads were shown to map to exonic regions and intergenic regions at similar rates [[12](#_ENREF_12), [13](#_ENREF_13)], suggesting lower confidence in measured expression below this level. Transcripts with an FPKM value below or equal to 0.3 were considered not expressed, so the threshold 0.3 FPKM is applied as the cut-off; (4) Filter based on the Coding Noncoding Index (CNCI, a powerful tool that effectively distinguishes protein-coding and non-coding sequences without known annotations): calculate the coding potential of each transcript using a local version of CNCI. CNCI has been proven to be effective for classifying incomplete transcripts, and especially well-suited to species that are not well-studied, because it can effectively classify transcripts solely based on the nucleotide composition of their sequence [[14](#_ENREF_14), [15](#_ENREF_15)], and; (5) Size selection: select multi-exonic transcripts longer than 200 bases.

**Publicly available reference and annotations.**

In this study, the reference Syrian golden hamster draft genome MesAur1.0 (ftp://ftp.ncbi.nlm.nih.gov/genomes/all/GCF_000349665.1_MesAur1.0/GCF_000349665.1_MesAur1.0_genomic.fna.gz) and the reference gene annotations (ftp://ftp.ncbi.nlm.nih.gov/genomes/all/GCF_000349665.1_MesAur1.0/GCF_000349665.1_MesAur1.0_genomic.gff.gz) were downloaded from NCBI Bioproject 77669 and 210213. And the known human and mouse lncRNAs were downloaded from NONCODE database. The known mouse and human miRNAs were downloaded from miRbase 21 (http://www.mirbase.org/).

**Reference**

1. Trapnell C, Pachter L, Salzberg SL. TopHat: discovering splice junctions with RNA-Seq. Bioinformatics. 2009;25(9):1105-11. doi: 10.1093/bioinformatics/btp120. PubMed PMID: 19289445; PubMed Central PMCID: PMC2672628.

2. Trapnell C, Roberts A, Goff L, Pertea G, Kim D, Kelley DR, et al. Differential gene and transcript expression analysis of RNA-seq experiments with TopHat and Cufflinks. Nature protocols. 2012;7(3):562-78. doi: 10.1038/nprot.2012.016. PubMed PMID: 22383036; PubMed Central PMCID: PMC3334321.

3. Grabherr MG, Haas BJ, Yassour M, Levin JZ, Thompson DA, Amit I, et al. Full-length transcriptome assembly from RNA-Seq data without a reference genome. Nature biotechnology. 2011;29(7):644-52. doi: 10.1038/nbt.1883. PubMed PMID: 21572440; PubMed Central PMCID: PMC3571712.

4. Haas BJ, Papanicolaou A, Yassour M, Grabherr M, Blood PD, Bowden J, et al. De novo transcript sequence reconstruction from RNA-seq using the Trinity platform for reference generation and analysis. Nature protocols. 2013;8(8):1494-512. doi: 10.1038/nprot.2013.084. PubMed PMID: 23845962; PubMed Central PMCID: PMC3875132.

5. Schulz MH, Zerbino DR, Vingron M, Birney E. Oases: robust de novo RNA-seq assembly across the dynamic range of expression levels. Bioinformatics. 2012;28(8):1086-92. doi: 10.1093/bioinformatics/bts094. PubMed PMID: 22368243; PubMed Central PMCID: PMC3324515.

6. Li W, Godzik A. Cd-hit: a fast program for clustering and comparing large sets of protein or nucleotide sequences. Bioinformatics. 2006;22(13):1658-9. doi: 10.1093/bioinformatics/btl158. PubMed PMID: 16731699.

7. Huang X, Madan A. CAP3: A DNA sequence assembly program. Genome research. 1999;9(9):868-77. PubMed PMID: 10508846; PubMed Central PMCID: PMC310812.

8. Huang da W, Sherman BT, Lempicki RA. Systematic and integrative analysis of large gene lists using DAVID bioinformatics resources. Nature protocols. 2009;4(1):44-57. doi: 10.1038/nprot.2008.211. PubMed PMID: 19131956.

9. Lu B, Zeng Z, Shi T. Comparative study of de novo assembly and genome-guided assembly strategies for transcriptome reconstruction based on RNA-Seq. Science China Life sciences. 2013;56(2):143-55. doi: 10.1007/s11427-013-4442-z. PubMed PMID: 23393030.

10. Kent WJ. BLAT--the BLAST-like alignment tool. Genome research. 2002;12(4):656-64. doi: 10.1101/gr.229202. Article published online before March 2002. PubMed PMID: 11932250; PubMed Central PMCID: PMC187518.

11. Martin JA, Wang Z. Next-generation transcriptome assembly. Nature reviews Genetics. 2011;12(10):671-82. doi: 10.1038/nrg3068. PubMed PMID: 21897427.

12. Ramskold D, Wang ET, Burge CB, Sandberg R. An abundance of ubiquitously expressed genes revealed by tissue transcriptome sequence data. PLoS computational biology. 2009;5(12):e1000598. doi: 10.1371/journal.pcbi.1000598. PubMed PMID: 20011106; PubMed Central PMCID: PMC2781110.

13. Hart T, Komori HK, LaMere S, Podshivalova K, Salomon DR. Finding the active genes in deep RNA-seq gene expression studies. BMC genomics. 2013;14:778. doi: 10.1186/1471-2164-14-778. PubMed PMID: 24215113; PubMed Central PMCID: PMC3870982.

14. Sun L, Luo H, Bu D, Zhao G, Yu K, Zhang C, et al. Utilizing sequence intrinsic composition to classify protein-coding and long non-coding transcripts. Nucleic acids research. 2013;41(17):e166. doi: 10.1093/nar/gkt646. PubMed PMID: 23892401; PubMed Central PMCID: PMC3783192.

15. Li P, Ruan X, Yang L, Kiesewetter K, Zhao Y, Luo H, et al. A Liver-Enriched Long Non-Coding RNA, lncLSTR, Regulates Systemic Lipid Metabolism in Mice. Cell metabolism. 2015;21(3):455-67. doi: 10.1016/j.cmet.2015.02.004. PubMed PMID: 25738460; PubMed Central PMCID: PMC4350020.
